# Beyond survival: growth trajectories in necrotizing enterocolitis

**DOI:** 10.1038/s41390-026-04765-3

**Published:** 2026-01-29

**Authors:** Parvesh Mohan Garg, Jeffrey Shenberger, Atul Malhotra

**Affiliations:** 1 Department of Pediatrics/Neonatology, Atrium Health Wake Forest Baptist, Wake Forest School of Medicine, Winston Salem, NC USA; 2 Department of Pediatrics/Neonatology, Connecticut Children’s, Hartford, CT USA; 3 Department of Pediatrics, Monash University, Melbourne, QLD Australia; 4 Monash Newborn, Monash Children’s Hospital, Melbourne, QLD Australia; 5 The Ritchie Centre, Hudson Institute of Medical Research, Melbourne, QLD Australia

Necrotizing enterocolitis (NEC) affects 5–10% of preterm neonates with a birth weight ≤1500 g.^1,2^ NEC remains a leading cause of mortality and morbidity due to triggering a systemic inflammatory response that induces multiorgan dysfunction and increases the risk of in-hospital mortality, generating a significant annual healthcare burden.^3^ A meta-analysis found that neurodevelopmental impairment occurs in as many as 40% of the infants with NEC, particularly among those requiring surgery.^4^ While cerebral palsy was the most frequent impairment reported, intraventricular hemorrhage also occurred more often in infants with NEC than in preterm infants without the disease.

A recent multicenter cohort study by Wang et al. enrolled 3046 infants born < 28 weeks' gestation from 2006 to 2019.^5^ Discharge growth trajectories expressed as z-scores of body weight (zBW) and head circumference (zHC) to corrected age 24 months were stratified by NEC severity (no/stage-I NEC, stage-II, stage-III) and epoch (1: 2006–2009; 2: 2010–2014; 3: 2015–2019) after propensity-score-matching. Over the 14 years reported, infants with stage-II or stage-III NEC had significantly lower mean zBW and zHC at discharge than those with no/stage-I NEC. By 24 months, the zBW were similar among the three groups, though the stage-III NEC group exhibited persistently lower zHC than infants in the no/stage I NEC group. A limitation of the study includes use of NEC staging rather than a surgical-based definition of NEC severity, thereby grouping infants together with known disparate outcomes. The study also did not examine post‑NEC nutritional practices—such as timing of feed reintroduction, fortification, or time to full feeds, factors which may influence growth outcomes independently of NEC itself.

A study by Hintz et al. found that surgical NEC was associated with significant growth delay and adverse neurodevelopmental outcomes at 18–22 months corrected age compared with infants without NEC.^6^ Although Ramel et al. reported that linear growth suppression in VLBW infants is negatively associated with developmental outcomes at 24 months,^7^ other studies note that NEC impacts neurodevelopmental outcomes without affecting growth in preterm infants at 18–24 months corrected age.^8,9^ A more recent report compared growth in preterm infants following surgical NEC to growth in infants with spontaneous intestinal perforation (SIP).^10^ The investigators found that infants with surgical NEC had significantly lower weight z scores at the time of discharge compared to infants with SIP. Specifically, no significant differences in weight, weight for length, head circumference, and length z scores before NEC/SIP onset, four weeks following NEC, and before anastomosis in the two groups were identified.^10^

The poor growth and impaired neurodevelopment in preterm infants with NEC may represent an increase in the level of pro-inflammatory cytokines compared to controls.^11^ Studies indicate that NEC is associated with white matter and cerebellar injury and accompanying brain volume loss, findings which translate into poor head growth at discharge and at 2-year follow-up. A recent study by Zhou et al. revealed that the brains of mice and humans suffering from NEC contain CD4^+^ T lymphocytes, cells integral to the development of brain injury.^12^ The authors postulate that gut-derived IFN-γ-releasing CD4^+^ T cells mediate neuroinflammation in infants with NEC by activating microglia, which in turn leads to loss of oligodendrocyte progenitors and myelin.^13^

Critically ill infants may also suffer poor growth secondary to low levels of insulin-like growth factor (IGF) -I and -II, as well as IGF binding protein-3 and accompanying elevated levels of growth hormone (GH), a consequence of developmental peripheral GH resistance.^14^ Infants with NEC, particularly those with stage-III disease, often face impaired nutrient absorption and gut-microbiota-brain axis dysregulation, which hinders head growth and growth in general, through the gut-brain cross talk.^15,16^ Poor weight gain postnatally also shares a known association with ROP.^17–19^ A recent study identified lower length, weight for length, and head circumference z scores at 36 weeks in infants with severe retinopathy of prematurity (ROP) with a history of NEC and SIP.^20^ Given that poor postnatal weight gain is linked to persistently low serum IGF-1 in preterm infants, it is conceivable that the poor weight gain experienced in infants with NEC is a marker for retinal IGF-1 insufficiency and the resultant aberrant vascular development of ROP.^21^

In summary, infants with surgical NEC are at greater risk for poor neurodevelopmental outcomes, poor somatic growth, and head growth at discharge and at 2 years of age. Studies that evaluate neuroprotective strategies to prevent brain injury will improve neurodevelopmental outcomes in high-risk preterm infants with NEC. Focusing clinical efforts on maintaining appropriate weight gain and linear growth during the critical window during which NEC and ROP develop will likely improve longer-term outcomes. By directing attention to early recognition of risk factors and biomarkers associated with brain injury following NEC, future studies may ultimately limit the life-long impact of NEC on neurodevelopment.
